# Integrated Transcriptomic Analysis Identifies Potential Biomarkers in Castration-Resistant Prostate Cancer

**DOI:** 10.14740/wjon2772

**Published:** 2026-06-25

**Authors:** Abdulghani A. Naeem, Saud A. Abdulsamad, Ateequllah Hayat, Ayesha Naeem, Nada Alhazmi, Ghaith Fallata, Anas Bokhari, Abdulmajeed H. Alharbi, Kinani A. Alkinani, Abdullah M. Alshehri, Khadijah M. Aldabbagh

**Affiliations:** a Department of Basic Sciences, College of Science and Health Professions, King Saud bin Abdulaziz University for Health Sciences, King Abdulaziz Medical City, Jeddah 21423, Saudi Arabia; b King Abdullah International Medical Research Center (KAIMRC), Jeddah 21423, Saudi Arabia; c Department of Molecular and Clinical Cancer Medicine, University of Liverpool, Liverpool L69 3PX, UK; d Department of Molecular and Biomedical Sciences, City St George’s, University of London, London, UK; e College of Medicine, King Saud bin Abdulaziz University for Health Sciences, Ministry of National Guard–Health Affairs, Jeddah 21423, Saudi Arabia

**Keywords:** Prostate cancer, Castration-resistant prostate cancer, RNA sequencing, Gene expression, Biomarkers, Bioinformatics

## Abstract

**Background:**

Castration-resistant prostate cancer (CRPC) represents an aggressive stage of prostate cancer that develops following resistance to androgen deprivation therapy. Although androgen receptor (AR) signaling remains a central driver of disease progression, additional adaptive molecular mechanisms contribute to therapeutic resistance. Understanding the transcriptional programs underlying CRPC may facilitate the identification of novel biomarkers and therapeutic targets.

**Methods:**

RNA sequencing–based transcriptomic profiling was performed to compare a non-malignant prostate epithelial model (PNT2) with a CRPC model retaining AR expression (22Rv1). Differential gene expression analysis was conducted using DESeq2. Functional enrichment analyses were performed using Gene Ontology (GO) and Kyoto Encyclopedia of Genes and Genomes (KEGG) pathway analyses. Gene co-expression network analysis was applied to identify coordinated regulatory interactions. Clinical validation of candidate genes was performed using Gene Expression Profiling Interactive Analysis 2 (GEPIA2), integrating datasets from The Cancer Genome Atlas (TCGA) and the Genotype-Tissue Expression (GTEx) project.

**Results:**

Transcriptomic comparison revealed extensive transcriptional remodeling associated with the castration-resistant phenotype. Several genes, including *MALAT1*, *FASN*, *PARP1*, *SET*, ENSG00000214719, and *IGF1*, were significantly dysregulated. Functional enrichment analyses demonstrated activation of metabolic processes, ribosome-associated pathways, nucleic acid binding functions, and PI3K–Akt signaling. Gene co-expression network analysis identified an AR-centered regulatory module involving *PARP1*, *KDM6B*, *BAZ2A*, *RANGAP1*, *NFAT5*, and *MAP4*. Clinical validation confirmed elevated expression of FASN, PARP1, SET, and ENSG00000214719 in prostate adenocarcinoma samples.

**Conclusions:**

Integrated transcriptomic and network analyses reveal coordinated metabolic, epigenetic, and DNA damage response pathways contributing to CRPC progression and identify potential combinatorial therapeutic vulnerabilities in advanced prostate cancer.

## Introduction

Prostate cancer (PCa) is a biologically heterogeneous disease that develops in the prostate gland, causing hyperplasia and malignancy in the prostatic tissues. PCa remains a major global health concern as it ranks as the most common diagnosed cancer in men worldwide [1, 2]. It is estimated that 67 per 100,000 people die from PCa every year in North America and Northern Europe [1]. The prevalence of PCa in Middle East has increased over the past 20 years by 125%, and the prevalence was 27 per 100,000 [3]. PCa is a multifactorial disease whose onset is influenced by several environmental and molecular factors including age, family history, race, genetic makeup, and immune system [2, 4]. The nuclear androgen receptor (AR) and its ligands play a central role in the initiation and progression of PCa, particularly during the early stages of the disease [5, 6]. Because PCa is largely androgen-dependent, first-line treatment typically involves androgen deprivation therapy (ADT) using anti-androgen agents such as bicalutamide, enzalutamide, or abiraterone acetate, with or without surgical castration [6–8]. Although ADT initially suppresses tumor growth, PCa frequently progresses to castration-resistant prostate cancer (CRPC), a lethal stage characterized by tumor adaptation and continued proliferation despite androgen-depleted conditions [6, 9, 10]. The progression of CRPC is driven by heterogeneous molecular mechanisms, including AR amplification, the emergence of constitutively active splice variants such as AR-V7, and *de novo* intratumoral androgen synthesis [10, 11]. Beyond AR signaling, progression is furthered by alternative oncogenic pathways, including PI3K/AKT and MAPK signaling, alongside neuroendocrine differentiation. Notably, metabolic deregulation has emerged as a crucial survival strategy [10, 12]. Recent research highlights the significance of fatty acid-binding protein 5 (FABP5) in CRPC, demonstrating that this lipid chaperone enhances tumor aggressiveness and facilitates a bypass of androgen dependence by activating the PPAR gamma and VEGF signaling axes [13]. Furthermore, FABP5 and related metabolic regulators contribute significantly to resistance against taxanes and next-generation anti-androgens [14]. Consequently, targeting the interplay between lipid metabolism and oncogenic signaling warrants further investigation to overcome therapeutic resistance in advanced PCa. To address these challenges, we performed RNA sequencing–based transcriptomic profiling to compare a non-malignant prostate epithelial model (PNT2) with a CRPC model retaining AR expression (22Rv1). Differential gene expression analysis was conducted using DESeq2, followed by Gene Ontology (GO) and Kyoto Encyclopedia of Genes and Genomes (KEGG) enrichment analyses to identify biological pathways associated with CRPC progression. To further explore regulatory relationships among dysregulated genes, a gene co-expression network analysis was performed to characterize network-level transcriptional coordination. Finally, the clinical relevance of candidate genes was evaluated using Gene Expression Profiling Interactive Analysis 2 (GEPIA2), integrating transcriptomic datasets from The Cancer Genome Atlas (TCGA) and the Genotype-Tissue Expression (GTEx) project. Identifying reliable molecular biomarkers and regulatory networks is essential for improving the diagnosis and management of CRPC and may support improved prognosis assessment and therapeutic targeting in advanced disease [15].

## Materials and Methods

### Cell lines and culture conditions

This study used two human prostate epithelial cell lines representing distinct biological states along the PCa continuum. PNT2 is a non-malignant prostate epithelial cell line derived from normal prostate tissue and immortalized using simian virus 40 (SV40) large T antigen, commonly used as a reference model for non-tumorigenic prostate epithelium [16]. 22Rv1 is a CRPC cell line established from a CWR22 xenograft that relapsed following androgen deprivation and is characterized by persistent AR expression, including both full-length AR and constitutively active splice variants, making it a widely used model of androgen-independent disease [16].

Both cell lines were obtained from the American Type Culture Collection (ATCC) and maintained under standardized culture conditions. Cells were grown as adherent monolayers in RPMI-1640 medium supplemented with 10% fetal bovine serum, L-glutamine, penicillin, and streptomycin, and incubated at 37 °C in a humidified atmosphere containing 5% CO_2_. To ensure experimental consistency and reduce technical variability, all experiments were conducted using three independent biological replicates, with cell lines maintained at comparable passage numbers and cultured using identical media formulations and seeding conditions [14].

### RNA extraction and library preparation

Total RNA was isolated from cryopreserved cell pellets using the RNeasy Mini Kit in accordance with the manufacturer’s instructions. RNA concentration and integrity were assessed using the Agilent TapeStation 4200, and only samples with an RNA integrity number (RIN) ≥ 8 were advanced for sequencing. To reduce ribosomal RNA content, samples underwent rRNA depletion using the Qiagen FastSelect rRNA HMR Kit prior to library construction. Sequencing libraries were then prepared using the NEBNext Ultra II RNA Library Prep Kit for Illumina. Library quantity was measured with a Qubit 2.0 Fluorometer, and library quality was further confirmed by quantitative polymerase chain reaction. High-throughput sequencing was carried out on the Illumina NovaSeq 6000 platform using a paired-end 2 × 150 bp read configuration (v1.5) to achieve an average depth of approximately 30 million paired-end reads per sample. Raw base call files were converted to FASTQ format and demultiplexed according to unique index sequences using bcl2fastq [11].

### Quality control and preprocessing

Sequencing read quality was assessed before and after preprocessing using FastQC; summary reports were generated through MultiQC. Adapter contamination and low-quality bases were removed using Trimmomatic by applying a sliding window filter (4 bp, Phred score ≥ 20), enforcing a minimum read length of 36 bp; and enabling adapter trimming. Only reads meeting quality thresholds following preprocessing were retained for downstream analyses [14].

### Transcriptomic data analysis

Filtered high-quality reads were aligned to the human reference genome (GRCh38) using the STAR aligner (version 2.7). Gene-level read counts were generated, and differential expression analysis was performed using the DESeq2 package (version 1.38) within the R statistical environment (version 4.2). Statistical significance was determined using the Benjamini–Hochberg false discovery rate (FDR) correction. Genes with an absolute log_2_ fold change ≥ 1 and an adjusted P value < 0.05 were considered significantly differentially expressed [13].

To interpret the functional relevance of the transcriptional changes, GO and KEGG enrichment analyses were performed to identify perturbed biological processes and signaling pathways. Enrichment analyses were conducted using an FDR-based adjustment method, and P-value and q-value cutoffs were set at 0.05. Enriched terms were derived from gene sets containing sufficient differentially expressed gene (DEG) representation, and results were reported using readable gene annotations. Functional enrichment analyses were performed using the clusterProfiler package in R, and significantly enriched pathways were identified using an FDR-adjusted P-value threshold of 0.05.

### Clinical validation using GEPIA2

Candidate genes emerging from differential expression and co-expression network analyses were further examined for clinical relevance using GEPIA2, a web-based resource integrating transcriptomic data from The Cancer Genome Atlas–Prostate Adenocarcinoma (TCGA-PRAD) and the GTEx project. Expression patterns of selected genes were assessed across tumor and normal prostate tissues to evaluate their association with PCa biology and to support the translational relevance of the identified gene signatures [15].

### Statistical analysis

Statistical evaluation of experimental data was performed using GraphPad Prism version 9. Depending on the experimental design, significance was determined using either two-tailed paired Student’s *t*-tests or one-way analysis of variance (ANOVA) followed by appropriate post hoc comparisons. All experiments were conducted with three independent biological replicates, and quantitative results are presented as mean ± standard deviation (SD) unless stated otherwise.

### Ethics approval

This study does not involve human participants or animal experiments; therefore, Institutional Review Board (IRB) approval was not required.

### Ethical compliance

This study was conducted in accordance with applicable institutional and international ethical standards.

## Results

### Differential transcriptomic landscape distinguishes PNT2 and 22Rv1 cells

**Figure 1:**
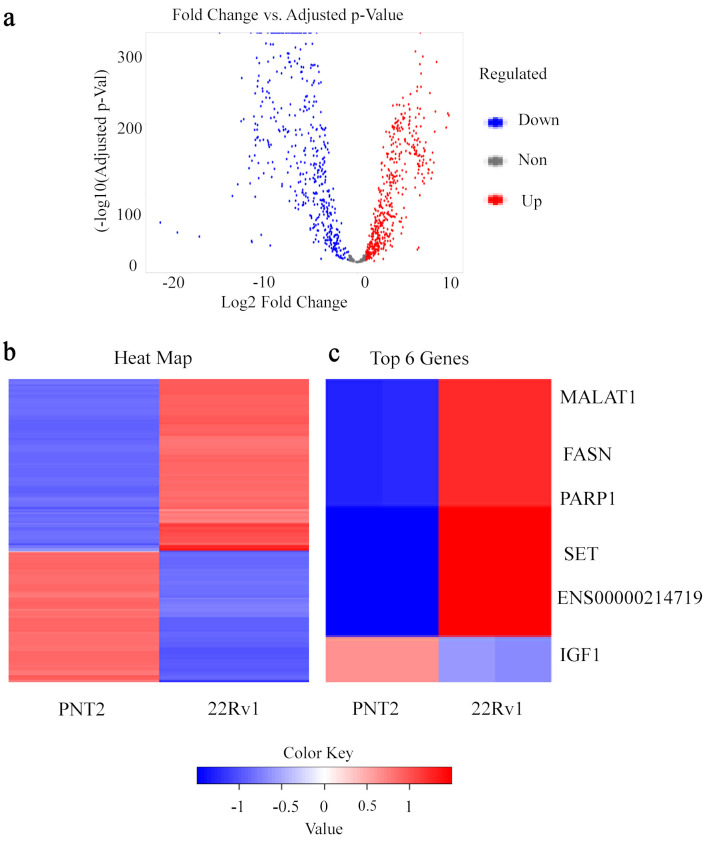
Differential transcriptomic landscape distinguishing PNT2 and 22Rv1 cells. (a) Volcano plot illustrating global differential gene expression between the non-malignant prostate epithelial cell line PNT2 and the castration-resistant prostate cancer cell line 22Rv1. Each point represents an individual gene distributed according to log_2_ fold change (x-axis) and statistical significance expressed as −log10 (adjusted P value) (y-axis). Genes significantly upregulated in 22Rv1 are highlighted in red, whereas genes downregulated relative to PNT2 are shown in blue. (b) Heatmap of the most significantly DEGs between PNT2 and 22Rv1 cells based on normalized transcript abundance. Hierarchical clustering reveals distinct gene expression patterns separating the non-malignant and castration-resistant cellular states. Red indicates relatively higher expression, whereas blue indicates lower expression. (c) Focused heatmap of representative genes identified among the top DEGs, including *MALAT1*, *FASN*, *PARP1*, *SET*, ENSG00000214719, and *IGF1*, illustrating clear transcriptional divergence between the two cellular models. DEGs: differentially expressed genes.

Figure 1 presents the global differential gene expression profile between PNT2 and 22Rv1 cells. The volcano plot (Fig. 1a) illustrates transcripts distributed according to log_2_ fold change and statistical significance, revealing extensive transcriptional remodeling associated with the castration-resistant phenotype. Differential expression analysis identified a substantial number of significantly altered transcripts between the two cellular states under the defined statistical thresholds. The most significantly DEGs are summarized in Table 1, including *MALAT1*, *FASN, PARP1*, *SET*, ENSG00000214719, and *IGF1*, which showed marked transcriptional differences between PNT2 and 22Rv1 cells. Heatmap visualization of these transcripts (Fig. 1b) demonstrates distinct expression patterns between PNT2 and 22Rv1, with coherent groups of genes exhibiting reciprocal expression across the two models. A focused view of representative genes (Fig. 1c) further highlights this divergence, where *MALAT1*, *FASN*, *PARP1*, *SET*, and ENSG00000214719 display increased expression in 22Rv1, whereas *IGF1* shows comparatively higher expression in PNT2. Together, these results demonstrate distinct transcriptional differences between PNT2 and 22Rv1 cells and provide a foundation for subsequent enrichment and network analyses.

**Table 1:** Top Differentially Expressed Genes Identified Between PNT2 and 22Rv1 Cells

Ensembl ID	Gene symbol	Log_2_ fold change	Adjusted P value (FDR)	Chromosomal location	Gene type
ENSG00000251562	*MALAT1*	17.60	1.34 × 10^–17^	11q13.1	lncRNA
ENSG00000169710	*FASN*	14.80	5.36 × 10^–17^	17q25.3	Protein-coding
ENSG00000143799	*PARP1*	11.24	9.67 × 10^–16^	1q42.12	Protein-coding
ENSG00000119335	*SET*	10.91	1.03 × 10^–15^	9q34.11	Protein-coding
ENSG00000214719	Unknown/lncRNA ENSG00000214719	10.23	1.88 × 10^–15^	17q11.2	lncRNA
ENSG00000174227	*IGF1*	−7.59	5.31 × 10^–14^	12q23.2	Protein-coding

ID: identifier; FDR: false discovery rate; lncRNA: long noncoding RNA.

### Biological process and cellular component enrichment analyses in PNT2 and 22Rv1 cells

**Figure 2:**
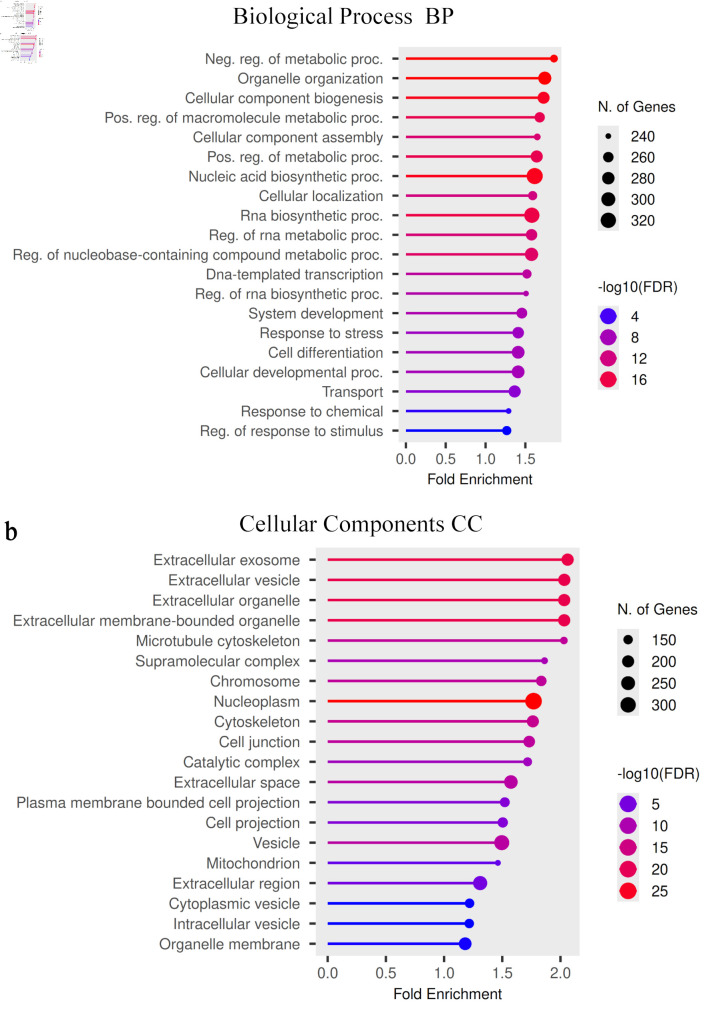
Gene Ontology (GO) enrichment analysis of differentially expressed genes. (a) GO biological process enrichment analysis of DEGs identified between PNT2 and 22Rv1 cells. The bubble plot displays significantly enriched biological processes associated with transcriptional remodeling in the castration-resistant state. The x-axis represents fold enrichment of each GO term. Bubble size indicates the number of genes contributing to each biological process, and color intensity represents statistical significance expressed as −log10(FDR). (b) GO cellular component enrichment analysis highlighting the subcellular compartments associated with the identified DEGs. Enriched categories include extracellular vesicle–related structures, cytoskeletal components, chromosomal elements, and mitochondrial-associated compartments. Bubble size reflects gene count, and color intensity represents −log10(FDR). FDR: false discovery rate; DEGs: differentially expressed genes. BP: biological process; CC: cellular component.

Figure 2 summarizes GO enrichment analysis of DEGs identified between PNT2 and 22Rv1 cells. A detailed list of the most enriched biological process terms is provided in Table 2.

**Table 2:** Gene Ontology (GO) Biological Process Enrichment Analysis of Differentially Expressed Genes Between PNT2 and 22Rv1 Cells

GO term	Biological process	FDR	Gene count	Pathway gene count	Fold enrichment
GO:0141187	Nucleic acid biosynthetic process	4.43 × 10^–19^	334	4,902	1.62
GO:0032774	RNA biosynthetic process	1.37 × 10^–16^	319	4,788	1.58
GO:0019219	Regulation of nucleobase-containing compound metabolic process	1.39 × 10^–14^	290	4,363	1.58
GO:0006996	Organelle organization	5.98 × 10^–20^	288	3,920	1.74
GO:0030154	Cell differentiation	1.03 × 10^–8^	284	4,782	1.41
GO:0048869	Cellular developmental process	1.03 × 10^–8^	284	4,783	1.41
GO:0009893	Positive regulation of metabolic process	6.42 × 10^–16^	276	3,988	1.64
GO:0006810	Transport	3.90 × 10^−7^	276	4,797	1.37
GO:0044085	Cellular component biogenesis	9.95 × 10^–19^	275	3,775	1.73
GO:0006950	Response to stress	3.21 × 10^–8^	270	4,545	1.41
GO:0051252	Regulation of RNA metabolic process	2.33 × 10^–13^	269	4,046	1.58
GO:0048731	System development	2.54 × 10^–9^	263	4,287	1.46
GO:0010604	Positive regulation of macromolecule metabolic process	6.42 × 10^–16^	259	3,659	1.68
GO:0051641	Cellular localization	1.10 × 10^–12^	251	3,743	1.59
GO:0006351	DNA-templated transcription	1.32 × 10^–10^	251	3,919	1.52
GO:0048583	Regulation of response to stimulus	4.67 × 10^–4^	251	4,704	1.27
GO:0009892	Negative regulation of metabolic process	7.50 × 10^–20^	244	3,115	1.86
GO:0022607	Cellular component assembly	9.49 × 10^–14^	241	3,468	1.65
GO:2001141	Regulation of RNA biosynthetic process	9.16 × 10^–10^	240	3,776	1.51
GO:0042221	Response to chemical	2.38 × 10^–4^	240	4,417	1.29

FDR: false discovery rate.

GO biological process enrichment (Fig. 2a) revealed significant overrepresentation of terms associated with regulation of metabolic processes, organelle organization, cellular component biogenesis, nucleic acid biosynthesis, RNA metabolic processes, and responses to cellular stimuli. These enriched categories reflect transcriptional changes related to metabolic regulation and macromolecular synthesis in 22Rv1 cells.

GO Cellular Component analysis (Fig. 2b) demonstrated significant enrichment in extracellular vesicle-related compartments, membrane-bounded organelles, microtubule cytoskeleton, chromosomes, cell junctions, and mitochondrial-associated structures. These enrichments reflect alterations in subcellular architecture and intercellular communication pathways associated with malignant transformation. The complete list of enriched cellular component categories is presented in Table 3.

**Table 3:** Gene Ontology (GO) Cellular Component Enrichment Analysis of Differentially Expressed Genes Between PNT2 and 22Rv1 Cells

GO term	Cellular component	FDR	Gene count	Pathway gene count	Fold enrichment
GO:0005654	Nucleoplasm	9.19 × 10^–28^	348	4,670	1.77
GO:0031982	Vesicle	2.70 × 10^–12^	288	4,571	1.50
GO:0005576	Extracellular region	1.74 × 10^–5^	258	4,675	1.31
GO:0005615	Extracellular space	3.67 × 10^–12^	240	3,619	1.57
GO:0031090	Organelle membrane	2.25 × 10^–2^	216	4,340	1.18
GO:0070062	Extracellular exosome	3.18 × 10^–22^	204	2,349	2.06
GO:0043230	Extracellular organelle	1.19 × 10^–21^	204	2,383	2.03
GO:0065010	Extracellular membrane-bounded organelle	1.19 × 10^–21^	204	2,383	2.03
GO:1903561	Extracellular vesicle	1.19 × 10^–21^	204	2,382	2.03
GO:0005856	Cytoskeleton	2.19 × 10^–14^	200	2,693	1.76
GO:0030054	Cell junction	6.39 × 10^–13^	190	2,605	1.73
GO:0005694	Chromosome	2.54 × 10^–13^	167	2,160	1.83
GO:0042995	Cell projection	1.36 × 10^–6^	163	2,572	1.50
GO:0120025	Plasma membrane bounded cell projection	1.30 × 10^–6^	157	2,449	1.52
GO:0031410	Cytoplasmic vesicle	2.79 × 10^–2^	151	2,941	1.22
GO:0097708	Intracellular vesicle	2.95 × 10^–2^	151	2,947	1.22
GO:1902494	Catalytic complex	2.74 × 10^–9^	144	1,990	1.72
GO:0015630	Microtubule cytoskeleton	2.53 × 10^–13^	131	1,532	2.03
GO:0099080	Supramolecular complex	2.29 × 10^–10^	127	1,618	1.86
GO:0005739	Mitochondrion	1.38 × 10^–4^	125	2,029	1.46

FDR: false discovery rate.

Together, these enrichment patterns indicate coordinated changes in metabolic regulation and structural organization that characterize the transcriptomic landscape of 22Rv1 cells relative to PNT2.

### Molecular function and KEGG enrichment analyses in PNT2 and 22Rv1 cells

**Figure 3:**
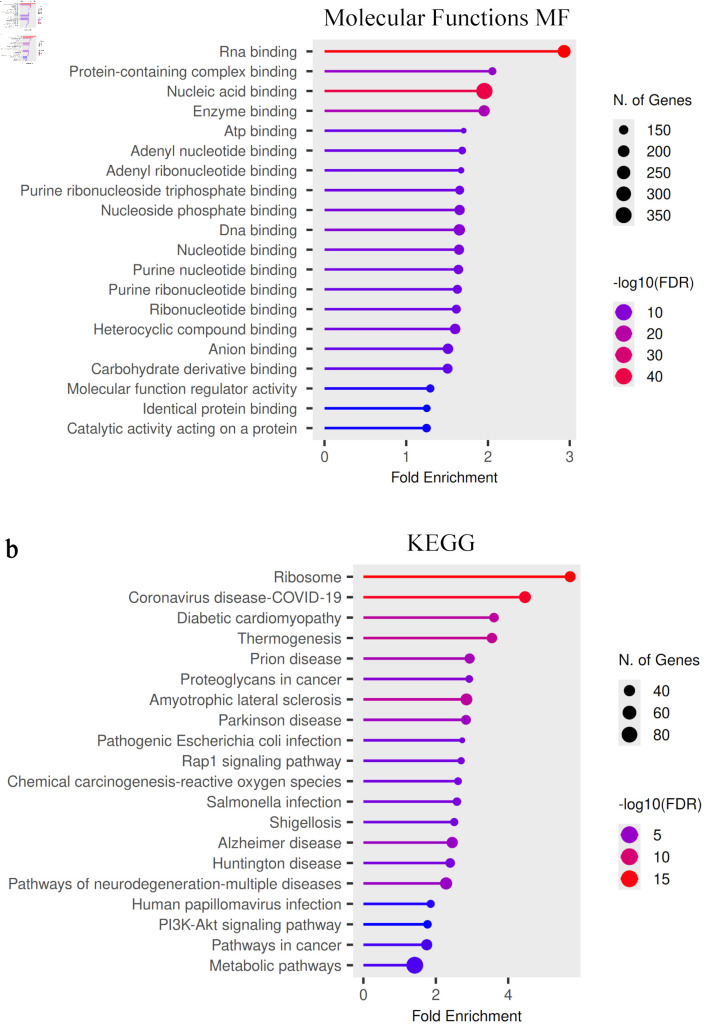
Functional enrichment analysis of molecular functions and signaling pathways. (a) Gene Ontology (GO) molecular function enrichment analysis of DEGs between PNT2 and 22Rv1 cells. Significantly enriched functions include nucleic acid binding, RNA binding, enzyme binding, and catalytic activity acting on proteins. These enrichments indicate increased activity of regulatory complexes and transcriptional control mechanisms in the castration-resistant phenotype. (b) KEGG pathway enrichment analysis identifying signaling pathways associated with the observed transcriptional changes. Significantly enriched pathways include ribosome-associated pathways, metabolic pathways, and PI3K–Akt signaling. Bubble size indicates the number of genes associated with each pathway, and color intensity represents statistical significance (−log10(FDR)). FDR: false discovery rate; DEGs: differentially expressed genes; MF: molecular function; KEGG: Kyoto Encyclopedia of Genes and Genomes.

Figure 3 illustrates the GO molecular function and KEGG pathway enrichment analyses of DEGs identified between PNT2 and 22Rv1 cells.

GO molecular function analysis (Fig. 3a) demonstrated significant enrichment in RNA binding, protein-containing complex binding, nucleic acid binding, enzyme binding, and catalytic activity acting on proteins. A comprehensive list of enriched molecular functions is summarized in Table 4. Enrichment of nucleotide and ribonucleotide binding functions further indicates extensive modulation of transcriptional regulation and post-transcriptional processing in the castration-resistant model. These findings suggest increased activity of macromolecular interaction networks and regulatory complexes that may contribute to altered gene expression control in 22Rv1 cells.

**Table 4:** Gene Ontology (GO) Molecular Function Enrichment Analysis of Differentially Expressed Genes Between PNT2 and 22Rv1 Cells

GO term	Molecular function	FDR	Gene count	Pathway gene count	Fold enrichment
GO:0003676	Nucleic acid binding	4.87 × 10^–40^	368	4,465	1.96
GO:0003723	RNA binding	2.53 × 10^–50^	233	1,886	2.93
GO:0003677	DNA binding	1.08 × 10^–10^	191	2,748	1.65
GO:0019899	Enzyme binding	2.01 × 10^–17^	189	2,296	1.95
GO:1901363	Heterocyclic compound binding	2.10 × 10^–8^	171	2,539	1.60
GO:0043168	Anion binding	8.33 × 10^–7^	170	2,671	1.51
GO:1901265	Nucleoside phosphate binding	3.28 × 10^–9^	167	2,398	1.65
GO:0000166	Nucleotide binding	5.51 × 10^–9^	165	2,380	1.65
GO:0097367	Carbohydrate derivative binding	2.75 × 10^–6^	160	2,521	1.51
GO:0017076	Purine nucleotide binding	4.43 × 10^–8^	152	2,202	1.64
GO:0032555	Purine ribonucleotide binding	1.78 × 10^–7^	144	2,102	1.63
GO:0032553	Ribonucleotide binding	2.56 × 10^–7^	144	2,119	1.61
GO:0035639	Purine ribonucleoside triphosphate binding	7.77 × 10^–8^	143	2,052	1.65
GO:0140096	Catalytic activity acting on a protein	3.40 × 10^–2^	138	2,624	1.25
GO:0098772	Molecular function regulator activity	1.31 × 10^–2^	134	2,457	1.29
GO:0044877	Protein-containing complex binding	2.66 × 10^–13^	132	1,525	2.05
GO:0030554	Adenyl nucleotide binding	1.66 × 10^–7^	129	1,817	1.68
GO:0042802	Identical protein binding	4.24 × 10^–2^	128	2,428	1.25
GO:0032559	Adenyl ribonucleotide binding	6.43 × 10^–7^	121	1,718	1.67
GO:0005524	ATP binding	2.82 × 10^–7^	120	1,673	1.70

FDR: false discovery rate; ATP: adenosine triphosphate.

KEGG pathway analysis (Fig. 3b) revealed significant enrichment in ribosome-associated pathways, metabolic pathways, and PI3K–Akt signaling. The full list of enriched pathways is provided in Table 5. Ribosome-associated pathways were among the most significantly enriched pathways identified in the analysis, while enrichment of metabolic and cancer-related pathways reflects reprogramming of cellular metabolism and survival signaling mechanisms. Additional enrichment in stress-related and infection-associated pathways likely reflects broader activation of adaptive cellular responses. Collectively, these data indicate coordinated alterations in protein synthesis, signaling cascades, and metabolic regulation in 22Rv1 cells compared with PNT2.

**Table 5:** KEGG Pathway Enrichment Analysis of Differentially Expressed Genes Between PNT2 and 22Rv1 Cells

KEGG pathway ID	Pathway name	FDR	Gene count	Pathway gene count	Fold enrichment
hsa01100	Metabolic pathways	3.13 × 10^–3^	93	1,556	1.42
hsa05022	Pathways of neurodegeneration–multiple diseases	6.50 × 10^–6^	46	478	2.28
hsa05171	Coronavirus disease 2019 (COVID-19)	7.96 × 10^–15^	44	234	4.46
hsa05014	Amyotrophic lateral sclerosis	3.77 × 10^–8^	44	367	2.85
hsa05010	Alzheimer disease	6.50 × 10^–6^	40	387	2.45
hsa05200	Pathways in cancer	3.98 × 10^–3^	39	529	1.75
hsa03010	Ribosome	3.63 × 10^–16^	38	158	5.71
hsa04714	Thermogenesis	7.77 × 10^–9^	35	234	3.55
hsa05020	Prion disease	1.10 × 10^–6^	34	275	2.93
hsa05012	Parkinson disease	5.53 × 10^–6^	32	268	2.83
hsa05415	Diabetic cardiomyopathy	3.79 × 10^–8^	31	204	3.61
hsa05016	Huntington disease	1.69 × 10^–4^	31	307	2.40
hsa05132	Salmonella infection	1.69 × 10^–4^	27	248	2.58
hsa04151	PI3K-Akt signaling pathway	1.59 × 10^–2^	27	362	1.77
hsa05131	Shigellosis	2.69 × 10^–4^	26	246	2.51
hsa05165	Human papillomavirus infection	1.07 × 10^–3^	26	332	1.86
hsa05205	Proteoglycans in cancer	5.16 × 10^–5^	25	203	2.92
hsa05208	Chemical carcinogenesis–reactive oxygen species	2.25 × 10^–4^	25	227	2.61
hsa04015	Rap1 signaling pathway	2.20 × 10^–4^	24	211	2.70
hsa05130	Pathogenic *Escherichia coli* infection	2.32 × 10^–4^	23	200	2.73

KEGG: Kyoto Encyclopedia of Genes and Genomes; ID: identifier; FDR: false discovery rate.

### Gene co-expression network analysis in PNT2 and 22Rv1 cells

**Figure 4:**
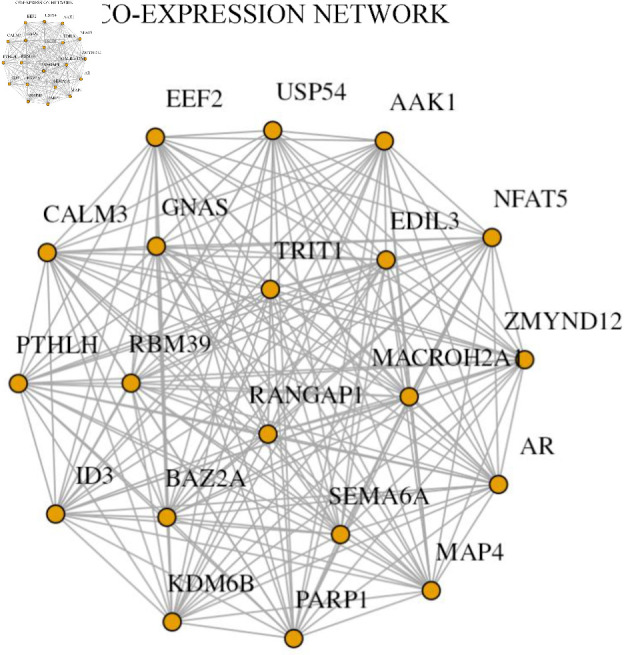
Gene co-expression network analysis of differentially expressed genes. The co-expression network was constructed from significantly differentially expressed genes identified in the transcriptomic comparison between PNT2 and 22Rv1 cells. Each node represents an individual gene, and edges represent significant correlation-based co-expression relationships. The network reveals a central regulatory module composed of *AR*, *PARP1*, *KDM6B*, *BAZ2A*, *RANGAP1*, *NFAT5*, and *MAP4*, which display multiple interaction links and high connectivity. The presence of this densely interconnected module highlights coordinated transcriptional regulation associated with the castration-resistant phenotype.

Figure 4 illustrates the gene co-expression network constructed from significantly DEGs identified between PNT2 and 22Rv1 cells. The network was generated based on significant pairwise expression correlations among the identified DEGs. The resulting architecture exhibits a densely interconnected structure, indicating coordinated expression patterns among genes deregulated in the castration-resistant 22Rv1 model relative to non-malignant PNT2 cells.

Within this network, a prominent core module comprising *AR*, *PARP1*, *KDM6B*, *BAZ2A*, *RANGAP1*, *NFAT5*, and *MAP4* emerged as highly connected nodes. These genes demonstrated multiple interaction links, suggesting coordinated transcriptional behavior in 22Rv1 cells. In contrast, such tightly interconnected relationships were less evident in the non-malignant context.

The AR occupied a central position within the network and displayed strong co-expression associations with several neighboring genes, including *PARP1*, which also exhibited a high degree of connectivity. Epigenetic regulators *KDM6B* and *BAZ2A* were closely linked to central nodes, while *RANGAP1*, *NFAT5*, and *MAP4* further contributed to the overall network structure. These genes formed a highly interconnected module within the co-expression network of DEGs.

Collectively, the co-expression network analysis reveals a structured transcriptional module that distinguishes 22Rv1 cells from PNT2 cells at the network level, complementing the differential expression and enrichment findings presented in previous figures.

### Clinical validation of selected candidate genes in prostate adenocarcinoma cohorts

**Figure 5:**
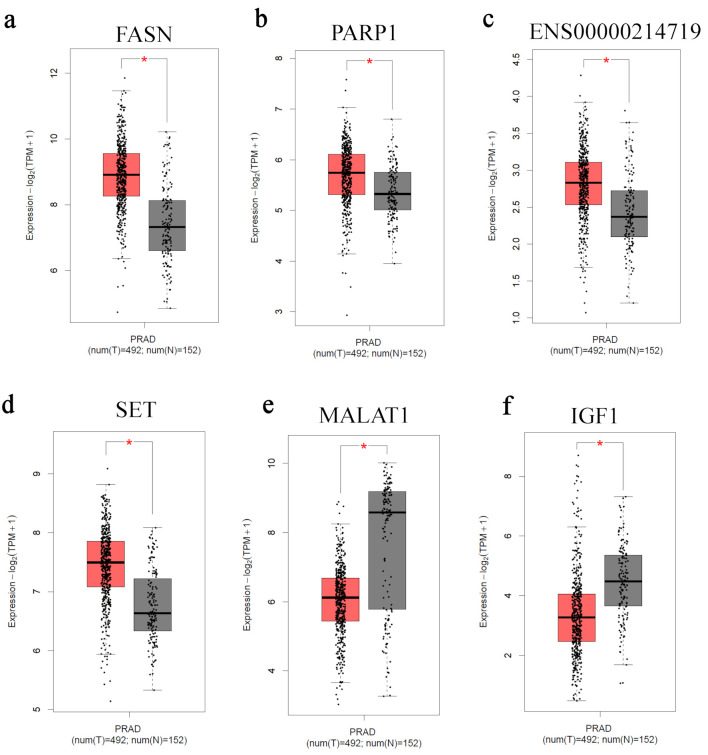
Clinical validation of candidate genes in prostate adenocarcinoma datasets. Boxplots showing the expression levels of (a) *FASN*, (b) *PARP1*, (c) ENSG00000214719, (d) *SET*, (e) *MALAT1*, and (f) *IGF1* in prostate adenocarcinoma (PRAD) tumors (n = 492) and normal prostate tissues (n = 152) obtained from The Cancer Genome Atlas (TCGA) and Genotype-Tissue Expression (GTEx) datasets via the Gene Expression Profiling Interactive Analysis 2 (GEPIA2) platform. Expression values are presented as log_2_(TPM + 1). The central line represents the median, the box indicates the interquartile range, and whiskers represent the data distribution. TPM: transcripts per million.

Figure 5 presents the clinical validation of selected DEGs using patient-derived prostate adenocarcinoma datasets from TCGA. Comparative analysis between tumor and normal prostate tissues demonstrated consistent alterations in gene expression patterns. *FASN*, *PARP1*, ENSG00000214719, and *SET* exhibited significantly higher expression levels in prostate adenocarcinoma samples relative to normal prostate tissues (Fig. 5a–d). In contrast, *MALAT1* and *IGF1* displayed reduced expression in tumor tissues compared with normal controls (Fig. 5e, f). These expression trends were consistent across the analyzed cohort and reached statistical significance. These findings support the potential clinical relevance of the identified genes as biomarkers in PCa.

## Discussion

### Metabolic reprogramming and translational adaptation in CRPC

Functional enrichment analyses revealed significant overrepresentation of metabolic processes and ribosome-associated pathways in 22Rv1 cells (Figs. 2, 3). The enrichment of ribosomal and RNA-binding molecular functions suggests enhanced translational capacity in the castration-resistant state [17]. Increased ribosome biogenesis and protein synthesis are well-recognized features of aggressive tumors [18], enabling rapid cellular proliferation and adaptation to therapeutic stress. In this context, activation of translational machinery may facilitate the production of proteins required for survival under androgen-deprived conditions [19]. Metabolic rewiring was further supported by the upregulation of fatty acid synthase (FASN), which was consistently elevated in 22Rv1 cells and was validated in clinical prostate adenocarcinoma samples (Fig. 5). FASN is a key enzyme responsible for *de novo* fatty acid synthesis and has been strongly associated with PCa progression [20]. Enhanced lipogenesis supports membrane biosynthesis and stabilizes signaling complexes that contribute to tumor growth and metabolic adaptation [21, 22]. The observed enrichment of PI3K–Akt signaling pathways further supports this model of metabolic–signaling integration [23]. PI3K–Akt activation has been widely implicated in resistance to ADT and often acts as a compensatory survival pathway when AR signaling is therapeutically suppressed [24, 25].

### Epigenetic regulation and chromatin remodeling in CRPC

Beyond metabolic and signaling adaptation, our network analysis revealed prominent involvement of chromatin-associated regulators within the transcriptional architecture of 22Rv1 cells. In the co-expression network (Fig. 4), *KDM6B* and *BAZ2A* emerged as highly connected nodes integrated within the *AR*-centered module, suggesting coordinated regulation of chromatin dynamics in the castration-resistant state [26, 27].

KDM6B (also known as JMJD3) is a histone demethylase that removes repressive H3K27me3 marks, thereby facilitating transcriptional activation. In PCa, epigenetic remodeling is increasingly recognized as a driver of lineage plasticity and therapeutic resistance. The integration of *KDM6B* within the core network identified in our study suggests that histone modification–mediated transcriptional reprogramming may contribute to the maintenance of *AR*-independent or *AR*-variant–driven gene expression programs in 22Rv1 cells. Similarly, *BAZ2A* is a chromatin remodeling factor implicated in transcriptional regulation and nucleolar organization [27]. Recent studies have associated *BAZ2A* with aggressive PCa phenotypes and epigenetic silencing of tumor suppressor pathways. Its strong co-expression with *AR* and other central nodes in our network supports the concept that chromatin accessibility and transcriptional plasticity are tightly coordinated with AR-associated signaling in CRPC [28].The enrichment of nucleic acid binding and RNA-related molecular functions observed in our GO analysis (Fig. 3a) further reinforces the role of epigenetic and transcriptional regulators in shaping the castration-resistant transcriptome. Together, these findings suggest that chromatin remodeling contributes to transcriptional plasticity that supports castration-resistant tumor adaptation [29].

### DNA damage response (DDR) adaptation and *PARP1*–*AR* crosstalk in CRPC

Our analysis identified *PARP1* as a prominently upregulated gene in 22Rv1 cells (Fig. 1) and as a highly connected node within the co-expression network (Fig. 4). Clinical validation further confirmed elevated *PARP1* expression in prostate adenocarcinoma samples relative to normal tissue (Fig. 5). These findings suggest that DDR adaptation is a central feature of the castration-resistant transcriptional program [30].

PARP1 plays a critical role in base excision repair and replication fork stabilization, enabling tumor cells to survive genotoxic stress [31]. In addition to its canonical DNA repair function, PARP1 has been shown to regulate AR transcriptional activity [32]. Prior studies have demonstrated that PARP1 interacts with AR and enhances AR-dependent gene expression, thereby linking DNA repair mechanisms to hormone-driven transcriptional regulation [33]. In AR-variant–expressing CRPC models such as 22Rv1, this functional coupling may allow tumor cells to sustain transcriptional output despite androgen deprivation [34]. PARP1 therefore appears to function at the intersection of DNA repair and AR–associated transcriptional regulation in CRPC [30].

Adaptive activation of the DDR has been associated with resistance to both AR pathway inhibitors and PARP inhibitors [35]. Restoration of homologous recombination, replication fork protection, and reduced PARP1 trapping are recognized mechanisms of therapeutic escape in advanced PCa. The elevated expression and central network positioning of PARP1 in our dataset suggest that DDR reinforcement may represent a compensatory mechanism contributing to the resilience of 22Rv1 cells [35].

### Growth factor–mediated bypass signaling and adaptive pathway compensation

Although *IGF1* expression was reduced in tumor tissues relative to normal prostate samples in the TCGA cohort (Fig. 5), the broader signaling context suggests a more complex role for IGF-mediated pathways in CRPC progression [36].

The enrichment of PI3K–Akt signaling pathways observed in our KEGG analysis (Fig. 3b) indicates activation of survival signaling networks in 22Rv1 cells. The insulin-like growth factor 1 receptor (IGF-1R) is a well-established upstream activator of PI3K–Akt and MAPK pathways, both of which contribute to proliferation, metabolic adaptation, and resistance to apoptosis [37]. Crosstalk between AR and IGF-1R signaling has been reported in PCa, where growth factor activation can partially compensate for reduced androgen signaling and sustain downstream oncogenic programs [38].

In castration-resistant models, bypass signaling through receptor tyrosine kinases and downstream kinase cascades may serve as an alternative mechanism to maintain survival when AR signaling is pharmacologically suppressed [39]. The coordinated enrichment of PI3K–Akt pathways, combined with metabolic rewiring and SET-mediated phosphatase inhibition discussed earlier, suggests that kinase-driven signaling reinforcement is a recurring feature of the CRPC transcriptomic landscape [40].The observed reduction in IGF1 transcript levels may reflect feedback regulation or altered ligand–receptor dynamics in advanced disease, rather than diminished pathway activity. In many hormone-driven cancers, receptor activation and downstream signaling intensity do not necessarily correlate directly with ligand transcript abundance, particularly in the context of tumor microenvironment-derived growth factors [41].

### Novel identification of ENSG00000214719 across distinct CRPC phenotypes

Among the DEGs identified in this study, ENSG00000214719 emerged as a consistently upregulated transcript in 22Rv1 cells and in prostate adenocarcinoma patient samples (Figs. 1, 5). Notably, in a separate ongoing investigation within our group, this long noncoding RNA (lncRNA) was also found to be highly expressed in DU145 cells, a CRPC model characterized by AR–negative status and glucocorticoid receptor–mediated signaling. The detection of ENSG00000214719 in both AR-positive (22Rv1) and AR-negative (DU145) castration-resistant models suggests that its expression may not be restricted to a single AR-dependent pathway. Instead, this lncRNA may represent a broader regulatory element associated with advanced, therapy-resistant PCa phenotypes. The presence of ENSG00000214719 across mechanistically distinct CRPC subtypes raises the possibility that it functions downstream of convergent resistance pathways or contributes to shared adaptive transcriptional programs. LncRNAs are increasingly recognized as regulators of chromatin remodeling, transcriptional control, and signaling pathway modulation [42, 43]. Given its recurrent upregulation in both AR-variant–expressing and AR-independent contexts, ENSG00000214719 may represent a previously underappreciated mediator of transcriptional plasticity in CRPC [34]. Its integration within the differential expression landscape identified in this study warrants further functional investigation to determine its role in drug resistance, lineage plasticity, or survival signaling. The consistent expression of ENSG00000214719 across distinct CRPC models represents a potentially novel observation, suggesting that this lncRNA may contribute to transcriptional plasticity associated with therapy-resistant PCa. The convergence of metabolic reprogramming, DDR activation, and chromatin remodeling highlights potential combinatorial therapeutic vulnerabilities beyond single-pathway targeting strategies.

### Integrated mechanistic signaling adaptation and therapeutic vulnerabilities in CRPC

The integrated transcriptomic, enrichment, co-expression, and clinical validation analyses collectively support a coordinated adaptive circuitry driving CRPC progression in 22Rv1 cells. Our results suggest that metabolic rewiring, kinase signaling activation, DDR adaptation, and AR-centered transcriptional regulation converge within an interconnected regulatory network driving CRPC progression (Figs. 2, 4). The consistent upregulation of FASN in both the experimental model and patient-derived tumors (Figs. 1, 5) implicates enhanced *de novo* lipogenesis as a structural and bioenergetic foundation of the resistant phenotype [44]. Increased fatty acid synthesis may stabilize membrane microdomains, facilitate receptor clustering, and potentiate downstream PI3K–Akt and MAPK signaling pathways, both enriched in our KEGG analysis (Fig. 3) [44]. In parallel, enrichment of ribosomal and mitochondrial-associated components (Figs. 2, 3) suggests heightened translational demand and metabolic flexibility, potentially supported by lipid-derived substrates fueling oxidative phosphorylation and adenosine triphosphate (ATP) generation [45]. Concurrently, elevated PARP1 expression and its central positioning within the co-expression network (Fig. 4), together with tumor-associated upregulation (Fig. 5), indicate reinforcement of DNA repair capacity integrated with transcriptional regulation [46]. PARP1 is known to modulate AR-driven gene expression, and in AR-variant–expressing systems such as 22Rv1, it may enhance ligand-independent transcriptional programs, thereby contributing to resistance against AR pathway inhibitors [47]. The enrichment of kinase signaling pathways despite reduced IGF1 transcript levels (Figs. 3, 5) further suggests network-level compensation mechanisms, potentially amplified by SET-mediated inhibition of PP2A and sustained downstream phosphorylation cascades [48]. Collectively, these findings support a coordinated regulatory network in which metabolic remodeling, kinase signaling activation, DDR adaptation, and epigenetic plasticity cooperate to sustain CRPC progression (Fig. 4) [34, 49]. This integrated circuitry provides a mechanistic explanation for persistent tumor growth under androgen deprivation. Importantly, the convergence of these adaptive axes highlights potential therapeutic vulnerabilities. Targeting lipogenic activation may disrupt signaling reinforcement at the membrane level, while inhibition of PARP1 could impair both DNA repair capacity and AR-associated transcriptional persistence. Modulation of chromatin remodeling enzymes may reduce transcriptional flexibility, and restoration of PP2A activity could attenuate hyperactive kinase signaling. The concordance between *in vitro* transcriptomic signatures and patient tumor expression profiles (Fig. 5) underscores the translational relevance of this network-based framework and supports the rationale for combination therapeutic strategies designed to disrupt multiple adaptive nodes simultaneously rather than targeting a single pathway in isolation.

### Study limitations

This study is based on *in vitro* transcriptomic profiling of established PCa cell lines with computational validation using publicly available clinical datasets. While 22Rv1 cells represent a well-characterized AR-variant–expressing CRPC model, they do not encompass the full molecular heterogeneity of advanced PCa. In addition, co-expression network analysis identifies coordinated transcriptional relationships but does not establish direct mechanistic causality. Functional validation in patient-derived models and *in vivo* systems will be required to confirm the regulatory roles of the identified candidate genes and to determine their contribution to therapeutic resistance.

### Conclusions

In conclusion, this study identifies key DEGs and transcriptomic alterations associated with CRPC. The integration of RNA sequencing and clinical validation data highlights several potential biomarkers, including *FASN*, *PARP1*, *SET*, and *MALAT1*, which may contribute to disease progression. These findings provide a basis for future functional studies and may support the development of diagnostic and therapeutic strategies in advanced PCa.

## Data Availability

The datasets generated and/or analyzed during the current study are available from the corresponding author upon reasonable request.

## Abbreviations

CRPC: castration-resistant prostate cancer
AR: androgen receptor
DEGs: differentially expressed genes
GO: Gene Ontology
KEGG: Kyoto Encyclopedia of Genes and Genomes
TCGA: The Cancer Genome Atlas
GTEx: Genotype-Tissue Expression
FDR: false discovery rate
